# Collaboration in Cancer Drug Trials (October 28-29, 2013): Abstracts

**DOI:** 10.1634/theoncologist.18-S1-1

**Published:** 2013-10

## Abstract 1 – EGFR-Mutant Lung Cancer: New Drugs

Lecia Sequist, Massachusetts General Hospital, Boston, Massachusetts, USA

The first-generation epidermal growth factor receptor (EGFR) tyrosine kinase inhibitors (TKIs; gefitinib, erlotinib) had a major impact on the treatment of non-small cell lung cancer and established the importance of early and universal genotyping for this disease. Many clinical obstacles remain, including acquired resistance and improvement of the duration of benefit. In the past year, we have seen the U.S. Food and Drug Administration approval of a second-generation EGFR TKI (afatinib) as well as the initial clinical reports of activity of third-generation EGFR TKIs. We will review the state of the art in EGFR-mutant lung cancer.

## Abstract 2 – The Role of Brachyury in Notochordal and Non-Notochordal Malignancy

Michael Kelley, Duke University Medical Center, Durham, North Carolina, USA

Brachyury is a T-box transcription factor, expressed in the notochord, that is essential for proper embryonic development. Brachyury is nearly universally expressed in chordoma, a notochord-derived tumor, and has been implicated as a human oncogene: humans carrying an extra germline copy of the brachyury gene are at high risk for chordoma. Brachyury is also expressed in 5% of other human cancers, and knockdown of brachyury in non-notochordal–derived tumor cells in vitro leads to growth arrest. The mechanism of activation of expression of brachyury in sporadic chordoma and non-notochordal tumors is uncommonly the result of gene duplication, and no somatically acquired mutations of brachyury have been described. Consequently, understanding of the pathways regulating brachyury expression may suggest tractable therapeutic targets. Expression analysis and chromosome immunoprecipitation have identified numerous putative transcriptional targets of brachyury, although none has yet been validated. Further investigation is ongoing to dissect those transcriptional targets that contribute to the malignant phenotype. A transcriptional reporter system has been designed for use in high-throughput screening to identify direct or indirect inhibitors of brachyury. Animal models of notochord and non-notochord targeted expression of brachyury are being developed.

## Abstract 3 – Chromatin Modulators Provide a New Insight Into Cancer Genomes

Johnathan Whetstine, Massachusetts General Hospital, Boston, Massachusetts, USA

Genomic instability is a hallmark of cancer that includes gains or losses of part or entire chromosomes. In fact, gains of chromosome 1q21 are affiliated with drug resistance and poor outcome in a number of malignancies. Therefore, understanding how these specific regions emerge and potentiate cancer will profoundly impact our understanding of hard-to-treat cancers. We recently demonstrated that the H3K9/36me3 demethylase KDM4A was amplified and overexpressed in multiple cancers, was co-amplified with specific sites such as 1q12-1q21, and was able to directly generate site-specific gains of these regions by disrupting heterochromatin. The KDM4A-dependent copy gains occurred within a single cell cycle, required S-phase, and are not stable but regenerated each cell division. KDM4A associated with replication machinery, which was enriched at KDM4A targets that undergo copy gain. Consistent with this observation, the copy gain regions underwent re-replication. We further demonstrated that the observed gains could be blocked by overexpressing the H3K9 methyltransferase Suv39h1/KMT1A or by overexpressing the heterochromatin binding protein HP1γ. Taken together, these data highlighted a role for chromatin state in regulating copy gains, which we further substantiated by generating copy gain with H3K9/K36 methylation interference. Therefore, our results demonstrate that overexpression of a chromatin modifier or alteration of certain chromatin states results in site-specific copy gains. These data begin to establish how copy number changes could originate during tumorigenesis and demonstrate that transient overexpression of chromatin modulators or the misregulation of the chromatin environment could promote copy change.

## Abstract 4 – New Lymphoma Therapies Inspired by Functional and Structural Genomics

Louis M. Staudt, Center for Cancer Genomics, Center for Cancer Research, National Cancer Institute, Bethesda, Maryland, USA

Seeking an unbiased method to discover therapeutic targets in cancer, we developed a loss-of-function genetic screen using genomic-scale libraries of small hairpin RNAs that mediate RNA interference. These “Achilles' heel” screens are designed to reveal genes essential for cancer cell proliferation and survival. In a parallel structural genomics approach, we are using RNA-seq to globally identify somatic mutations and other structural abnormalities in cancer. The intersection of these two data sets has helped us to discover novel pathogenetic pathways in the most common type of non-Hodgkin lymphoma, diffuse large B-cell lymphoma (DLBCL). The ABC DLBCL subtype has constitutive activation of the NF-κB pathway, which we traced to the signaling adapter CARD11 using the Achilles' heel screen. In approximately 10% of ABC DLBCL tumor biopsies, we discovered recurrent CARD11 mutations that spontaneously activate NF-κB signaling. We also defined a “chronic active” form of B-cell receptor (BCR) signaling that activates NF-κB in ABC DLBCLs with wild type CARD11. Such ABC DLBCLs are killed by knockdown of BCR signaling components, such as the kinase BTK or components of the BCR itself. Over one fifth of ABC DLBCLs have mutations affecting the CD79B or CD79A subunits of the BCR. In 18% of cases, mutations occur in a single tyrosine residue in the critical “ITAM” signaling motif, generating BCRs that avoid negative autoregulation by the LYN tyrosine kinase. Our Achilles' heel screens also revealed that ABC DLBCLs depend on MYD88, a key adapter in Toll receptor signaling. RNA-Seq uncovered somatic mutations in the MYD88 TIR domain in 39% of ABC DLBCLs with a single point mutation (L265P) accounting for 29% of cases. Genetic evidence suggests an interplay between the BCR and MYD88 signaling pathways in a subset of ABC DLBCL tumors. To attack chronic active BCR signaling therapeutically, we have initiated clinical trials in relapsed/refractory DLBCL of ibrutinib, an irreversible and highly selective small molecule inhibitor of BTK. Thus far, ibrutinib monotherapy has induced a high rate of partial and complete responses, the vast majority of which are in ABC DLBCL. Responses have been observed in “primary refractory” tumors that had never responded to any prior therapy. Within ABC DLBCL, recurrent mutations in the BCR and MYD88 pathways explain some, but not all, differences in response to ibrutinib. Given its excellent safety profile and selective mechanism of action, ibrutinib can be combined rationally with both chemotherapy and other signaling modulators to achieve cures for these patients.

## Abstract 5 – Protein Methyltransferase Inhibitors as Personalized Cancer Therapeutics

Robert A. Copeland, Epizyme, Inc., Cambridge, Massachusetts, USA

The protein methyltransferases (PMTs) constitute a class of enzymes that catalyze the methylation of lysine or arginine residues on histones and other proteins. A number of PMTs have been shown to be genetically altered in cancers through, for example, gene amplification, chromosomal translocations, point mutations, and synthetic lethal relationships. The enzymes DOT1L and EZH2 provide two representative examples of altered PMTs that act as genetic drivers of specific human cancers. The enzymatic activity of DOT1L is associated with a chromosomal translocation that is universally found in patients with MLL-rearranged leukemia. Point mutations at Y641 of EZH2 are found in a subset of non-Hodgkin lymphoma patients; the enzymatic activity of both wild-type and mutant EZH2 are required for pathogenesis in these patients. In addition, deletion of the INI1 (SMARCB1 or hSNF5) subunit of the SWI/SNF chromatin-remodeling complex occurs in nearly all malignant rhabdoid tumors (MRTs), a childhood cancer with a particularly poor prognosis. An antagonistic relationship has been demonstrated between the biochemical action on chromatin of the SWI/SNF complex and EZH2 (in the context of the polycomb repressive complex 2) that is relieved in MRTs because of the INI1 deletion. Consequently, MRTs demonstrate increased reliance on the enzymatic activity of EZH2 for proliferation, and we have shown that MRT cells deficient in INI1 are selectively killed by inhibition of EZH2 in cell culture and in mouse xenograft models. Drug discovery efforts have yielded potent, selective inhibitors of each of these targets that have now transitioned into phase I clinical trials. These inhibitors affect the appropriate histone methyl marks in cells, lead to selective cell killing that is dependent on genetic alteration of the target enzyme, and affect tumor growth inhibition in xenograft models. These data portend the effective use of selective PMT inhibitors as a novel modality for future personalized cancer therapeutics.

## Abstract 6 – Histone H3K27 Demethylases as a Therapeutic Target in Polysomy 21 Leukemia

Andrew Lane, Dana-Farber Cancer Institute, Boston, Massachusetts, USA

Polysomy 21 is highly associated with B-cell acute lymphoblastic leukemia (B-ALL), but the mechanisms underlying this association are unclear. We analyzed hematopoiesis and leukemogenesis in the Ts1Rhr mouse model that triplicates 31 genes orthologous to the Down syndrome critical region (DSCR). DSCR triplication caused B-cell differentiation abnormalities, conferred self-renewal to B-cell progenitors, and cooperated with BCR-ABL to promote B-ALL. Gene expression in DSCR-triplicated B cells and polysomy 21 B-ALL patients was associated with upregulation of genes normally repressed by the polycomb repressor complex 2 (PRC2). PRC2 is responsible for catalyzing the methylation of histone H3K27, a post-translational modification associated with gene silencing. Histone mass spectrometry confirmed that B cells and B-ALLs with DSCR triplication have H3K27 hypomethylation, consistent with loss of PRC2 function. Small molecule inhibition of EZH2 (the catalytic subunit of PRC2) was also sufficient to confer H3K27 hypomethylation and self-renewal properties to B-cell progenitors. We hypothesized that restoring H3K27 methylation by inhibiting histone demethylases might block transformation associated with DSCR triplication. Treatment with GSK-J4, a small molecule inhibitor of the H3K27 demethylases UTX and JMJD3, blocked the growth of B-cell progenitors with DSCR triplication. Murine B-ALLs were sensitive to GSK-J4 in the low-micromolar range, whereas B-ALLs with DSCR triplication were 2–3 logs more sensitive. Human B-ALLs with and without trisomy 21 were also sensitive to GSK-J4 treatment. Consequently, restoration of histone H3K27 methylation by inhibition of H3K27 demethylases may be an effective treatment for B-ALL, particularly in cases with polysomy 21.

## Abstract 7 – Bacterial Genomics in Transplant-Related Colitis

Ami S. Bhatt, Dana-Farber Cancer Institute, Boston, Massachusetts, USA

Significant and treatment-limiting morbidity and mortality are associated with the administration of cancer chemotherapy. Patients who have clear tumor responses to chemotherapy are not infrequently subjected to dose reductions or significant changes of otherwise efficacious regimens because of known “side effects” of the drugs, infectious and immunologic complications, and/or complications resulting from an unknown cause—so-called idiopathic syndromes. Only a small fraction of the effort that has been expended to improve cancer outcomes has been focused on reducing treatment-related morbidity and mortality. This work investigates the central hypothesis that a subset of highly morbid cancer-treatment–related idiopathic syndromes are triggered by heretofore unidentified microbial pathogens. The sequencing-based discovery of *Bradyrhizobium enterica*, a potential stem cell transplantation-associated pathogen, has been described, using a genomics-based approach. Given the ease, rapidity, and culture-independent method of discovery of a disease-associated bacterium that was demonstrated in the previously idiopathic cord colitis syndrome, it is anticipated that this methodology will be more widely applied for the identification of potential disease-associated pathogens. Discussion will address the technological approach used to identify this organism as well as the potential implications of this work for clinicians and translational researchers in the fields of infectious diseases, hematology, and oncology.

## Abstract 8 – “Click” Chemistry In Vivo: New Tools for Molecular Imaging

Jonathan Carlson, Massachusetts General Hospital, Boston, Massachusetts, USA

One of the outstanding challenges in clinical medicine is the accurate detection of diseases at the earliest stages of their natural history. This work requires resolutions far below what is currently achievable, on the millimeter rather than the centimeter or multicentimeter scale. Indeed, it is at the sub-cubic millimeter scale that cancers are least likely to have metastasized and thus are most curable by resection; just as pivotally, occult metastatic disease, hiding below the limit of detection, is the central player in both adjuvant therapy and late relapse. Recognizing this critical biological threshold, the NIH has highlighted the need for new detection technology as one of its provocative challenges. New molecular and physical methods will be required to meet this goal—to increase signal strength and sensitivity, to enhance biological contrast (i.e., to identify and exploit molecular characteristics that distinguish normal tissue from abnormal tissue), and to enhance spatial resolution. We have recently described a set of fluorescent dyes that combines exceptionally efficient on/off switching capabilities, becoming more than 1,500-fold brighter on activation, with the ability to chemically react with a labeled target molecule in vivo. These probes harness “click” chemistry, through the exceptionally rapid and specific reactivity of a class of molecules known as “tetrazines,” for superb target-to-background ratios, and they open the door to new mechanisms for molecular imaging and intravital diagnostics.

## Abstract 9 – Leveraging Genetically Engineered Mouse Models of Small Cell Lung Cancer for Advanced Therapeutic Studies

Anna Farago, Dana-Farber Cancer Institute and Massachusetts General Hospital Cancer Center, Boston, Massachusetts, USA

Small cell lung cancer (SCLC) is among the most lethal solid tumor malignancies, yet treatment outcomes have remained virtually unchanged for 30 years. New treatment paradigms are sorely needed. We have developed a series of genetically engineered mouse models of SCLC, in which conditional deletion of the tumor suppressor genes *p53* and *Rb1* in pulmonary epithelial cells triggers initiation of tumors that mirror the histology, molecular features, genetic alterations, and metastatic potential of human SCLC. A subset of tumors also inactivates a third tumor suppressor gene, *Pten*, resulting in increased PI3K pathway signaling. Using in vitro and in vivo systems, we are exploring the use of targeted therapies for treating SCLC. Strategies under investigation include inhibition of the PI3K signaling pathway and combination target of rapamycin complex (TORC) and Bcl-2 family protein inhibition. We find that although PI3K pathway inhibitors are active in cell lines derived from murine SCLC tumors, inhibition of PI3K pathway signaling only minimally slows the growth of tumors in vivo; however, combining TORC inhibition with the inhibition of the antiapoptotic Bcl-2 family proteins results in potent cell death and significant tumor regression in vivo in some tumors. Using genetically engineered mice as a preclinical system, we are exploring these and other targeted therapies to develop better treatment strategies for patients with SCLC.

## Abstract 10 – TERT Promoter Mutations in Cancer

Franklin Huang, Dana-Farber Cancer Institute, Boston, Massachusetts, USA

Systematic genomic sequencing studies of human cancers have identified many mutations in coding regions of the genome. We sought to examine whether recurrent mutations occurred in the noncoding region of the genome, using whole-genome sequencing data from human melanomas. We found that 71% of melanomas contained either one of two independent mutations in the promoter of the TERT gene. Each of these mutations created identical consensus ETS transcription factor binding sites. Cancer cell lines and other cancer types also contain these TERT promoter mutations at high frequency. These data suggest that TERT promoter mutations and noncoding mutations in general may be common oncogenic events in human cancer.

## Abstract 11 – Next Steps in Melanoma Treatment

Keith Flaherty, Massachusetts General Hospital, Boston, Massachusetts, USA

BRAF inhibitors and immune checkpoint inhibitors have transformed the treatment landscape in melanoma. Single-agent BRAF inhibitors and CTLA4 blockade are giving way to BRAF/MEK combined therapy and PD1/PDL1 antibodies. Refining the application of these therapies requires a focus on predictive biomarkers to understand which patients derive the greatest benefit. It is also critical to understand the mechanisms of de novo and acquired resistance to develop rational combination therapies and to develop them in an individualized fashion.

## Abstract 12 – Multiple Roles of MITF

David E. Fisher, Massachusetts General Hospital, Boston, Massachusetts, USA

Microphthalmia-associated transcription factor (MITF) is a transcription factor that is essential to melanocyte development and functions as a master regulator of pigmentation. MITF is also an amplified or mutated oncogene in a fraction of melanomas, where it modulates pathways central to survival, metabolism, and differentiated state (i.e., melanoma subtype). Multiple signaling pathways have been identified that modulate MITF's expression or biochemical activity. Some of these, like the melanocyte stimulating hormone pathway, mediate ultraviolet-induced pigmentation (sun tanning). Recent data suggest that byproducts of the ultraviolet pathway are also molecular mediators of altered behavior. This same pathway also regulates basal pigmentation (e.g., fair skin). Several molecular strategies have been investigated that may alter expression or activity of MITF within melanocytes or melanoma cells. The elucidation of such approaches may lead to novel regulators of pigmentation (skin cancer risk) or represent novel approaches to melanoma treatment.

## Abstract 13 – Molecular Analysis of CTCs

David Tsai Ting, Massachusetts General Hospital, Boston, Massachusetts, USA

Circulating tumor cells (CTCs) are shed from primary tumors into the bloodstream, mediating the hematogenous spread of cancer to distant organs. Using a mouse model of pancreatic cancer, we applied a microfluidic device to isolate very rare CTCs independently of tumor epitopes, subjecting these to single-cell RNA sequencing. This has provided unprecedented transcriptomic resolution of these cells and has identified key pathways that are important in metastasis. This has provided the groundwork for using RNA signatures in CTCs as a potential pharmacodynamics biomarker and has opened new avenues to discover novel therapeutics.

## Abstract 14 – Molecular Regulation of Normal and Malignant Stem Cells

George Daley, Children's Hospital Boston, Boston, Massachusetts, USA

LIN28, a developmental regulator originally identified as a heterochronic mutant in *Caenorhabditis elegans*, functions as an RNA binding protein that regulates protein expression through effects on mRNA translation and through control of processing of the *let-7* family of tumor suppressor microRNAs. Lin28 has two paralogs, Lin28A and Lin28B, and studies have revealed a role for these factors in a wide array of biological phenotypes, including organismal growth and metabolism, sexual maturation, and oncogenesis. LIN28/let-7 act antagonistically to regulate the balance of self-renewal and differentiation in embryonic stem cells, and LIN28 is one of the factors shown to reprogram somatic cells to pluripotency. In pediatric cancers, high-level expression of LIN28 is a consistent marker of germ cell tumor. It is frequently observed in neuroblastoma and hepatoblastoma and activated by chromosomal translocation in Wilms tumor. By immunohistochemistry, Lin28 is overexpressed in a wide array of solid tumors, including ovarian and colon cancer. By transgenic overexpression of Lin28 in mice, we have generated a variety of murine models of cancer involving the kidney, liver, gut, soft tissues, and blood. Thus, Lin28/let-7 represents a novel pathway in a significant percentage of human cancers, and understanding its downstream effectors and its role in tumor maintenance will define its value as a prognostic and therapeutic target.

## Abstract 15 – CTC Developments

Richard Lee, Massachusetts General Hospital, Boston, Massachusetts, USA

Prostate cancer commonly metastasizes to bone. Obtaining tissue from bone metastases to gain molecular insights into disease progression is limited by low yield. Current standards for evaluation of post-treatment response (serum prostate-specific antigen, bone scan, abdominal/pelvic computed tomography) also have limitations. Improved biomarkers are needed. Circulating tumor cells (CTCs) are shed by primary and metastatic cancers. Improved capture of CTCs from peripheral blood has been reported using multiple approaches. Clinical applications of CTCs could include, but are not limited to, enumeration of CTCs in response to therapy, identification of CTCs as a diagnostic test, or detailed molecular characterization of CTCs during the course of therapy. This discussion will describe the current and potential future uses of CTCs in prostate cancer management with a focus on microfluidic techniques developed at the Massachusetts General Hospital Cancer Center. The advent of such “liquid biopsies” could provide noninvasive sampling to direct targeted therapies in advanced prostate cancer.

## Abstract 16 – Neoadjuvant Therapy

Mary-Ellen Taplin, Dana-Farber Cancer Institute, Boston, Massachusetts, USA

At diagnosis, localized prostate cancer is categorized into low-, intermediate-, and high-risk groups based on clinical stage, baseline prostate specific antigen, and biopsy Gleason score. Although many men are cured with local therapy, including prostatectomy and radiation, the cure rate for some intermediate-risk and high-risk patients is suboptimal. Historically, neoadjuvant therapy with androgen deprivation did not improve relapse rates. The neoadjuvant trials conducted in the 1990s were hampered by lack of patient stratification and by short durations of androgen deprivation with weak androgen depleting agents. The development of new approaches to androgen deprivation therapy, including CYP17 lyase inhibitors (abiraterone) and androgen signaling inhibitors (enzalutamide), has provided rationale for re-evaluating neoadjuvant androgen deprivation combined with prostatectomy. Data will be presented demonstrating the prostate tissue effects of the combination abiraterone/lupron compared with lupron and the prostatectomy.

## Abstract 17 – Novel Agents in Advanced Disease

Matthew R. Smith, Massachusetts General Hospital, Boston, Massachusetts, USA

The treatment landscape for metastatic prostate cancer has been transformed in recent years by the approval of multiple therapies that increase overall survival. These recently approved therapies include a cell-based immunotherapy, a second-generation taxane, a bone-seeking radiopharmaceutical, a small molecule targeting the androgen receptor (AR), and a drug that targets androgen biosynthesis. Despite these important advances, there are many unmet medical needs, including greater understanding of the optimal timing, sequencing, and combination of available agents and the development of new agents to overcome mechanisms of resistance. This presentation will focus on emerging therapies for castration-resistant prostate cancer, including next-generation drugs that target the AR and/or androgen biosynthesis and new targeted therapies, including small molecule inhibitors of vascular endothelial growth factor and MET.

